# Analysis of single nucleotide polymorphisms of *CRYGA* and *CRYGB* genes in control population of western Indian origin

**DOI:** 10.4103/0301-4738.49393

**Published:** 2009

**Authors:** Suman Kapur, Shipra Mehra, Devarshi Gajjar, Abhay Vasavada, Manav Kapoor, Shashwat Sharad, Bhagwat Alapure, S Rajkumar

**Affiliations:** Biological Sciences Group, Birla Institute of Technology and Science, Pilani – 333 031, Rajasthan, India; 1 Iladevi Cataract and IOL Research Centre, Gurukul Road, Memnagar Ahmedabad - 52, Gujarat, India

**Keywords:** Cataract, γ-crystallins, Indian, polymerase chain reaction, restriction fragment length polymorphism, single nucleotide polymorphism

## Abstract

**Aim:**

Polymorphisms in γ-crystallins (*CRYG*) can serve as markers for lens differentiation and eye disorders leading to cataract. Several investigators have reported the presence of sequence variations within crystallin genes, with or without apparent effects on the function of the proteins both in mice and humans. Delineation of these polymorphic sites may explain the differences observed in the susceptibility to cataract observed among various ethnic groups. An easier Restriction Fragment Length Polymorphism (RFLP)-based method has been used to detect the frequency of four single nucleotide polymorphisms (SNPs) in *CRYGA*/*CRYGB* genes in control subjects of western Indian origin.

**Materials and Methods:**

A total of 137 healthy volunteers from western India were studied. Examination was performed to exclude volunteers with any ocular defects. Polymerase chain reaction (PCR)-RFLP based method was developed for genotyping of G198A (Intron A), T196C (Exon 3) of *CRYGA* and T47C (Promoter), G449T (Exon 2) of *CRYGB* genes.

**Results:**

The exonic SNPs in *CRYGA* and *CRYGB* were found to have an allele frequency 0.03 and 1.00 for ancestral allele respectively, while frequency of non-coding SNP in *CRYGA* was 0.72. Allele frequency of T90C of *CRYGB* varied significantly (*P* = 0.02) among different age groups. An *in-silico* analysis reveals that this sequence variation in *CRYGB* promoter impacts the binding of two transcription factors, ACE2 (Member of CLB2 cluster) and Progesterone Receptor (PR) which may impact the expression of *CRYGB* gene.

**Conclusions:**

This study establishes baseline frequency data for four SNPs in *CRYGA* and *CRYGB* genes for future case control studies on the role of these SNPs in the genetic basis of cataract.

Crystallins (*CRY*) are the dominant structural components of the vertebrate eye lens having a two-domain beta structure, folded into four very similar Greek key motifs,[1] which help in maintaining lens transparency. Several cataract-causing mutations have been identified in the γ-crystallins (*CRYG)* genes both in mouse and man.[2] Mutations in these genes implicate the *CRYG* gene cluster as a very critical locus for lens development and differentiation. In a review, Graw *et al.,*[2] listed a variety of polymorphic sites that have been identified in the mouse *Cryga* and *Crygb* genes and showed that some mutations occurring in these genes were associated with different cataract phenotypes. Recently Li *et al.,*[3] reported that a point mutation occurring in the *Crygb* gene in a mouse causes dominant dense nuclear cataract. Rogaev *et al.,*[4] studied a tri-nucleotide microsatellite marker for gamma-crystallin B gene (*CRYG1*) and found it to co-segregate with polymorphic congenital cataract (PCC) yielding a maximum LOD score of 10.62. Santhiya *et al.,*[5] also reported that the variation G198A of Intron A in *CRYGA* occurred at a fairly high frequency in cases of autosomal dominant cataract cases. In addition to this, the allele – T47C is found to affect the promoter of the *CRYGB* gene and occurs in five out of 10 cases in a heterozygous condition in family studies.[5] The *CRYGC* and *CRYGD* genes have been extensively studied in humans while the potential role of *CRYGA* and *CRYGB* still remains to be ascertained. Thus, for the current study these two genes were chosen for establishing the baseline frequency in western Indians. Information on polymorphic sites in cataract-related genes in the affected and unaffected population, at large, may explain the genetic predisposition to cataract and also the underlying genomic diversity in different ethnic groups. The present study was the first Polymerase Chain Reaction (PCR)-Restriction Fragment Length Polymorphism (RFLP)-based approach to screen certain single nucleotide polymorphisms (SNPs) at a population level in order to obtain a baseline frequency for use in future case-control studies.

## Materials and Methods

A total of 137 unrelated healthy volunteers comprising 90 males, 47 females (age range 2.5–67 years) who visited the local eye hospital for an annual eye checkup during the period May 2005 to December 2006 were recruited for the study. The study was approved by the Institutional Ethical Review Committee (IERC). A subject qualified as a control if (a) both the pupils could be dilated to at least 6 mm, (b) both lenses were graded as having no nuclear, posterior sub-capsular, cortical opacities including Grade I or II opacities. Venous peripheral blood samples were collected from the subjects after obtaining an informed consent. Genomic DNA was extracted from the collected samples using a standard protocol.[6]

Primer sequences as reported by Santhiya *et al.,*[5,7] were used for amplification of the target regions by PCR. The obtained amplicon was divided into two parts and while one part was digested with appropriate restriction endonuclease the other undigested part was used as reference to compare with the fragments generated after digestion with the restriction endonuclease. Specific restriction endonucleases (procured from Fermentas) were used to study the restriction site affected by the reported nucleotide variations based on the restriction maps generated using New England Biolabs (NEB) cutter software.[8] The digested and undigested PCR products were analyzed using 12% Polyacrylamide Gel Electrophoresis (PAGE) in 1XTBE. Table 1 lists the scheme of restriction endonuclease used and the DNA fragments obtained after digestion of PCR amplicon with respective restriction endonuclease at conditions as per the manufacturer's guidelines (incubation at 37°C for *Nmu*CI, *Hae*III and *Pst*I, and 65°C for *Taq*I overnight). All PCR-RFLP-based analysis was confirmed with DNA sequencing in representative cases.

**Table 1:** Restriction endonucleases used to detect various polymorphisms in *CRYGA* and *CRYGB*

Gene	PCR Amplicon		SNPs	Restriction endonuclease used	Size of fragments (bp) obtained after restriction endonuclease digestion in different genotypes*		
						
	Region	bp					
CRYGA	Intron A	463	G198A	*Nmu*CI	GG	AA	GA
							
			(rs796280)		197, 193, 73	270, 193	270, 197, 193, 73
	Exon 3	355	T196C	*Hae*III	TT	CC	TC
							
					305, 50	160, 145, 50	305, 160, 145, 50
CRYGB	Promoter	519	T47C	*Psf*I	TT	CC	TC
							
			(rs2289917)		426, 93	519	519, 426, 93
	Exon 2	519	G449T	*Taq*I	GG	TT	GT
							
					519	450, 69	519, 450, 69

**Figure 1:**
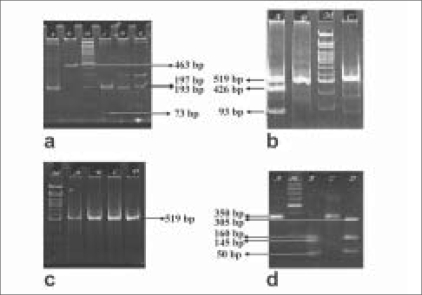
Restriction fragments obtained for different polymorphisms studied in *CRYGA* and *CRYGB* genes (a) Band profile obtained after digestion with *NmuCI (CRYGA*, INTRON A, G198A) (b) Band profile obtained after *Pst*I digestion: (*CRYGB* PROMOTER T47C) (c) Band profile obtained after digestion with *Taq*I (*CRYGB* EXON 2, G449T) (d) Band profile obtained after *Hae*III digestion (*CRYGA*, EXON3 T196C)

* Figure 1; PCR - Polymerase chain reaction, SNP - single nucleotide polymorphism, bp - base pairs

Allele frequencies were estimated by allele counting method and differences in frequencies between the two age groups were determined using two-way contingency table and Chi square test. Hardy-Weinberg estimates were performed using the Michael Court online calculator. The putative changes in the transcription factor binding sites were studied, using AliBaba software[9] that scans for potential transcription factor binding sites, for sequence variations in the promoter region of *CRYGB* gene.

## Results

Four SNPs, namely G198A and T196C in Intron A and Exon 3 of *CRYGA*, T47C in promoter and G449T in Exon 2 of *CRYGB* were studied and the sequence variations could be easily identified on the basis of the restriction fragments obtained in each case as evident from the gel images shown in Fig. 1. The observed genotype frequencies satisfy Hardy Weinberg Equilibrium for all polymorphisms studied Tables 2 and 3. Out of 137 volunteers, 40% were found to be heterozygous for G198A *CRYGA* polymorphism (frequency of “A” allele = 0.28) [Table 2]. The frequency of 196C allele in Exon 3 of *CRYGA* was found to be very high (0.97). Analysis for T47C polymorphism in promoter region of *CRYGB* revealed that 1.5% subjects were homozygous for TT; 66.2% subjects were homozygous for CC and the remaining 32.3% subjects were TC heterozygous [Table 2]. A significant difference was observed (*P*=0.02) when the frequency of 47T allele was compared in subjects stratified for age [Table 3]. As the frequency of TT allele was found to be the same in all subjects above the age of 10 years all further analysis was done using the age stratification of <12 years and >12 years of age which is the norm for segregating pediatric and adult cases in the medical profession.[10,11] The allele frequency for “T” was 0.23 in <12 year olds and 0.11 in those >12 years Tables 3 and 4. No sequence variation was observed at Nucleotide 449 in Exon 2 of *CRYGB* as all 121 subjects analyzed were found to have “GG” genotype [Table 2]. The allele frequencies obtained were compared with frequencies reported for other populations worldwide [Table 5] and are significantly different from those reported by Santhiya *et al.,*[5,7] in families with history of autosomal dominant congenital cataract.

**Table 2:** Distribution of genotypes and alleles in the western Indian population

Gene	Single nucleotide polymorphism		Genotype numbers			Allele frequency		n
CRYGA	Intron A G198A (rs796280)	GG	AA	GA	G	A	
							
		71	11	55	0.72 (197)	0.28 (77)	137
			HWE (p = 0.99)				
CRYGA	Exon 3 T196C	TT	TC	CC	T	C	
							
		00	03	42	0.03 (03)	0.97 (90)	45
			HWE (p = 1)				
CRYGB	Promoter T47C (rs2289917)	TT	CC	TC	T	C	
							
		02	88	43	0.18 (47)	0.82 (219)	133
			HWE (p = 0.43)				
CRYGB	Exon 2 G449T	GG	TT	GT	G	T	
							
		121	00	00	1.00 (242)	0.00 (00)	121
			HWE (p = 1)				

HWE - Hardy Weinberg equilibrium

**Table 3:** Distribution of genotypes and alleles for *CRYGB* T47C (promoter) single nucleotide polymorphism

Age (in years)	Mean age ± SD (in years)	Genotype numbers			Allele frequency		2n	*P* value
					
		CC	TC	TT	C	T		
00–10	5.45 ± 2.6	34	28	2	0.76 (70)	0.24 (32)	128	
			HWE (*P* = 0.1 8)					
11–20	13.65 ± 2.3	19	05	0	0.89 (43)	0.11 (5)	48	0.01^1^
			HWE (*P* = 0.56)					
21–30	24.88 ± 2.9	14	03	0	0.91 (31)	0.09 (3)	34	0.81^2^
			HWE (*P* = 0.68)					
31–40	35 ± 2.8	05	01	0	0.92 11	0.08 (1)	12	0.96^3^
			HWE (*P* = 0.82)					
41–50	47 ± 3.5	02	01	0	0.83 (5)	0.17 (1)	6	0.59^4^
			HWE (*P* = 0.72)					
51–60	54.9 ± 2.7	08	01	0	0.94 (17)	0.06 (1)	18	0.39^5^
			HWE (*P* = 0.86)					
>60	63.6 ± 2.9	02	03	0	0.70 (7)	0.30 (3)	10	0.22^6^
			HWE (*P* = 0.33)					
>11	29.66 ± 17.7	50	14	0	0.89 (114)	0.11 (14)	128	0.0002^7^
			HWE (*P* = 0.83)					

SD - Standard deviation, HWE - Hardy weinberg equilibrium,

“1” = *P* value between age group 0–10 and 11–20 years,

“2” = *P* value between age group 11–20 and 21–30 years,

“3” = *P* value between age group 21–30 and 31–40 years,

“4” = *P* value between age group 31–40 and 41–50 years,

“5” = *P* value between age group 41–50 and 51–60 years,

“6” = *P* value between age group 51–60 and >60 years,

“7” = *P* value between age group 0–10 and >11 years

**Table 4:** Distribution of genotypes and alleles for *CRYGB* T47C (promoter) single nucleotide polymorphism

Gene	Single nucleotide polymorphism	Age (years)	Genotype numbers			Allele frequency		n
CRYGB	Promoter T47C (rs2289917)		TT	CC	TC	T	C	
								
		≤12	02	42	30	0.23* (34)	0.77 (114)	74
				HWE *P* = 0.45)				
		>12	00	46	13	0.11* (13)	0.89 (105)	59
				HWE (*P* = 0.63)				

χ^2^(Yates' corrected CHI square) = 5.66, *P* = 0.02, HWE - Hardy weinberg equilibrium

**Table 5:** Comparison of obtained allele frequencies with frequency reported in different populations

World populations	n	*CRYGA* (Intron A) 198A (rs796280)	n	*CRYGB* (Promoter) 47C (rs2289917)	Reference
CEPH	30	0.155	120	0.642	SNP database, NCBI
Yoruba, Nigeria	30	0.254	120	0.942	
Caucasian	-	-	90	0.560	
African American	-	-	92	0.870	
Han Chinese	45	0.522	88	0.811	
Tokyo, Japan	44	0.440	90	0.852	
Yusuke, Japan	752	0.581	752	0.581	
Western Indian	137	0.280#	133	0.820*	Present study (2008)
Southern Indian	19	0.470#	23	0.557*	Santhiya *et al*., (2002, 2004)

# (Odds ratio = 7.1, 95% confidence interval = 1.57–31.9);

* (Odds ratio = 22.5, 95% confidence interval = 3.7–135.4);

CEPH-Utah residents with ancestry from northern and western Europe

**Figure 2:**
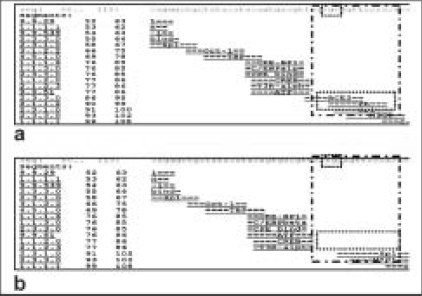
Effect of T47C variation of *CRYGB* on transcription factor binding sites: Comparison between the most common 47C (a) and the rare allele 47T (b) for the putative transcription factor binding sites by AliBaba Version 2.1 software. The large dotted box shows the sites of alteration with corresponding transcription factors (small dotted box indicates sites of single nucleotide polymorphism and the transcription factors binding)

The sequence variation in *CRYGB* promoter region was also analyzed for change in transcription factor binding sites using the AliBaba software. While the sequence containing the “C” allele at nucleotide position 47 has binding sites for transcription factor ACE2 and PR, the substitution by “T” at this position results in the loss of both these binding sites [Fig. 2].

## Discussion

Crystallins in lens do not turn over and must serve the lens for the lifetime of a person. Thus, the lens is even more dependent than most other tissues on protection from any kind of damage. Besides maintaining lens transparency, βγ-crystallins (Beta and gamma crystallins), may also function as stress protection proteins that are induced during periods of critical stress on the retina.[1] Sequence changes occurring in the form of nucleotide polymorphisms in these protective systems could critically lead to accumulation of abnormally folded proteins eventually leading to disease.[12,13] γ-crystallins may also have developmental roles and numerous SNPs in their genes have been linked to hereditary cataracts. Santhiya *et al.*,[5,7] have reported a co-segregation of SNPs in *CRYG*, *CRYBB2* and *GJA8* genes with familial congenital cataract. At the same time there are other contradictory reports both in mice and in humans on polymorphic sites within these genes with no apparent effects on the function of the respective proteins.[14,15]

In the present study, the first of its kind in India, the baseline frequency for four SNPs in *CRYGA* and *CRYGB* genes has been studied in healthy Indian volunteers with no history of any eye disease including cataract. A review of literature reveals that 12 years is the given norm for categorization of patients into pediatric cases.[10,11] The observed difference in the allele distribution of *CRYGB* promoter region, T47C, with age may be due to the inherent inability to exclude the subjects susceptible to age-related cataract from the younger group (<12 years of age) while all such individuals in the older group would have been excluded from the study due to the rigorous exclusion criteria followed during this study. Selection of “control or unaffected” population is an important aspect of case-control design for studying genetic markers for age-related disorders. Therefore special attention must be paid to patient/subject recruitment as certain nucelotide changes may play a critical role during perinatal and/or paediatric growth phase only.

It is also interesting to note that the same SNP when analyzed for putative transcription factor binding site (through AliBaba software) shows altered binding for two transcription factors. While the T47 allele looses the binding site for transcription factors ACE 2 and PR, the 47C allele retains both these binding sites. This is an important finding in the light of reports showing that progesterone leads to glucocorticoid-like effects in various tissues[16,17] and the long-term use of glucocorticoids induces cataract. As the *CRYGB* gene has not been characterized well in humans,[3] our observation on the putative alteration of transcription factor binding sites warrants future studies to delineate the specific role of this allele in the etiology of eye disorders and disease progression.

When the frequencies obtained in the present study were compared with those reported in different populations of the world by NCBI SNP database (dbSNP),[18] the allele frequency for 198G→A in *CRYGA* gene was found to be similar to that observed in Africans. Frequency of T47C in the promoter region of *CRYGB* is similar to those reported for Africans, Chinese and Japanese. It is noteworthy that allele frequencies for both these polymorphisms differ within Japanese sub-populations, emphasizing the fact that differences do exist within a geographical region.[19] No database records are available for frequencies of *CRYGA* T196C (Exon 3) and *CRYGB* G449T (Exon 2). The observed frequency for T196C is similar to that reported earlier by Santhiya *et al.,*[5] in congenital cataract cases, indicating that this polymorphism may have no role in cataractogenesis. When the present findings (in healthy volunteers) are compared with the incidence of these polymorphisms in cataract probands studied by Santhiya *et al.,*[5,7] a significant difference in the frequency of *CRYGA* 198A (Odds ratio = 7.1, 95% CI = 1.57−31.9) and *CRYGB* 47C mutation (Odds ratio = 22.5, 95% CI = 3.7−135.4) is observed, implicating a role of these mutations in cataractogenesis. The *CRYGC* and *CRYGD* genes are already well studied in case of humans, and the potential role of *CRYGA* and *CRYGB* is yet to be explored. The current study establishes the baseline frequency for specific sequence variations in *CRYGA-B* genes which will be useful for future case-control studies in this ethnic group. It has yet to be experimentally proved that functional promoter variation in *CRYGB* and non-coding variant of *CRYGA* may affect expression or generate splice variants of *CRYG* genes. These findings will give insights into genetics of cataract/s. These kinds of studies will be of paramount importance in order to guide development of a medical therapy that will prevent or delay the onset of adult cataract, lessening the burden on the aging population and the consequent requirement for large numbers of surgical procedures. The present study needs to be extended in cataract patients to ascertain the association with the etiology of cataractogenesis.
